# Effects of product visual design consistency on brand recognition and state anxiety: a psychophysiological study integrating heart rate variability and self-report measures

**DOI:** 10.3389/fpsyg.2026.1841820

**Published:** 2026-08-11

**Authors:** Yinqiu Yang, Peng Huang

**Affiliations:** 1 School of Art and Design, Bengbu University, Bengbu, Anhui, China; 2 Faculty of Medical Psychology, Air Force Medical University, Xi’an, China

**Keywords:** brand recognition, heart rate variability, product visual design consistency, psychophysiology, state anxiety, user experience

## Abstract

**Background:**

Product visual design consistency is regarded as an important characteristic of product family design and brand identity. In industrial design, it is understood as the overall coherence of multiple visual attributes across products within a product family. Although such coherence has been suggested to facilitate information processing and product recognition, its potential psychophysiological associations with consumer responses remain insufficiently understood. As an exploratory investigation, this study aims to provide preliminary evidence of the psychophysiological correlates of visual design consistency in real-world commercial contexts.

**Methods:**

This exploratory study employed a within-subject design to examine systematic associations between visual design consistency and multiple response domains. A within-subject experiment was conducted with 64 university students. Participants completed brand identification tasks using standardized images of two commercial product systems representing relatively high and relatively low levels of visual design consistency. During the tasks, electrocardiography (ECG) was continuously recorded to derive heart rate variability (HRV) indices (SDNN and RMSSD), while brand recognition accuracy and State-Trait Anxiety Inventory-State (STAI-State) scores were also collected.

**Results:**

Compared with products characterized by relatively lower visual design consistency, products with relatively higher visual design consistency were associated with significantly higher brand recognition accuracy (*p* < 0.001), higher SDNN (*p* = 0.016, *r* = 0.31), higher RMSSD (*p* < 0.001, *r* = 0.61), and lower STAI-State scores (*p* = 0.002, *r* = 0.39). The three response domains (behavioral performance, HRV indices, and self-reported anxiety) showed convergent patterns across conditions, indicating systematic co-occurrence of differences in cognitive performance, autonomic activity, and subjective emotional states between the two product systems.

**Conclusion:**

These findings provide exploratory psychophysiological evidence that exposure to commercial product systems characterized by relatively higher visual design consistency is systematically associated with differences in brand recognition performance, autonomic nervous system activity, and state anxiety. As an exploratory investigation employing ecologically valid commercial product systems in which multiple visual attributes and brand-related characteristics naturally co-occur, the findings should be interpreted as evidence of multimodal associations rather than causal effects. Future studies using experimentally controlled stimuli are warranted to disentangle the respective contributions of individual visual design dimensions and related perceptual factors.

## 1 Introduction

### 1.1 Practical context and origin of the research question

In contemporary consumer markets, product differentiation is increasingly achieved not only through functional innovation but also through the systematic construction of visual identity across product families. As product ecosystems expand in scale and complexity, manufacturers frequently rely on coherent visual languages to signal brand identity and facilitate product recognition across heterogeneous product lines. Within industrial design research, such coherence is commonly discussed under the concept of visual design consistency, which refers to the perceived coordination of multiple visual attributes across products belonging to the same brand or product family (Bloch, 1995; Crilly et al., 2004).

Unlike narrow interpretations that focus solely on single visual dimensions such as shape or color, design research conceptualizes visual design consistency as a holistic perceptual construct integrating multiple interrelated attributes, including form language, chromatic scheme, material expression, proportion, detailing, and overall stylistic coherence (Mugge et al., 2015; Veryzer and Hutchinson, 1998). From a brand perspective, such perceptual coherence may also function as a signal of product family consistency, thereby shaping perceived brand extension fit and category-based evaluations (Aaker and Keller, 1990; Park et al., 1991). From this perspective, visual consistency is not reducible to any single feature but emerges from the coordinated structure of a product system as a whole.

In parallel with the evolution of product ecosystems, increasing attention has been given to how visual design characteristics influence consumer perception, including product preference, brand evaluation, and recognition performance. Prior research has shown that more prototypical or visually coherent designs are generally associated with higher aesthetic preference and easier cognitive processing (Crilly et al., 2004; Veryzer and Hutchinson, 1998). However, most existing studies have primarily relied on behavioral ratings or self-report measures, with relatively limited integration of physiological indicators that reflect autonomic and affective responses during real-time product perception.

More recently, consumer neuroscience and neurodesign research have begun to address this gap by incorporating psychophysiological measures such as electroencephalography (EEG), eye-tracking, and heart rate variability (HRV) to investigate consumer responses to visual and aesthetic stimuli (Plassmann et al., 2015; Reimann et al., 2010). Among these measures, HRV has been widely used as an index of autonomic nervous system regulation and cognitive-affective processing under different perceptual and decision-making conditions (Shaffer and Ginsberg, 2017).

### 1.2 Conceptualization of visual design consistency in product systems

Building on this theoretical background, visual design consistency in the present study is conceptualized as a multidimensional construct reflecting the degree of perceptual coherence across multiple visual attributes within a product system. Rather than being limited to a single feature dimension, it is understood as an emergent property of coordinated design decisions that jointly shape the overall visual impression of a brand’s product portfolio.

Accordingly, consistent product systems are typically characterized by stable and unified visual patterns across form language, color scheme, material expression, proportional structure, detail treatment, and stylistic identity. Conversely, lower-consistency systems tend to exhibit greater heterogeneity across these visual dimensions, resulting in less stable perceptual integration at the system level.

Importantly, this construct-oriented definition is grounded in established industrial design theory, where visual coherence is considered a key mechanism through which product families communicate brand identity and facilitate consumer recognition across diverse product categories (Bloch, 1995; Crilly et al., 2004). In this sense, visual design consistency should be understood as a holistic perceptual property of real product systems rather than a manipulation of isolated visual features.

This systemic perspective is particularly relevant when studying real-world commercial products, where multiple visual attributes co-occur naturally and cannot be fully separated without compromising ecological validity.

We acknowledge that employing real commercial systems entails a trade-off between ecological validity and experimental control over individual low-level visual features. Our design intentionally prioritizes the former, and consequently, our findings should be interpreted as reflecting the aggregate effect of naturally co-occurring visual attributes, rather than the isolated causal contribution of formal consistency alone. This framing is explicitly considered throughout the Discussion.

### 1.3 From behavioral evaluation to psychophysiological evidence in consumer research

Although prior research has established important links between visual design characteristics and consumer responses, most empirical findings have been derived from explicit evaluations such as preference ratings, recognition accuracy, or attitude measures. While informative, these approaches may not fully capture the underlying cognitive and affective processes that occur during real-time exposure to product stimuli.

To address this limitation, consumer neuroscience has introduced psychophysiological methodologies that enable the measurement of autonomic and neural responses during product perception. In particular, HRV has been widely applied as a non-invasive index of autonomic nervous system activity, reflecting the dynamic balance between sympathetic and parasympathetic regulation during cognitive and affective processing (Shaffer and Ginsberg, 2017).

Recent studies suggest that visual complexity, perceptual fluency, and aesthetic coherence may be associated with differential autonomic responses, potentially reflected in HRV indices (Reimann et al., 2010; Vecchiato et al., 2014). However, empirical evidence linking real-world product system-level visual coherence to simultaneous behavioral, physiological, and affective responses remains limited.

### 1.4 Research gap and study objectives

Despite growing interest in visual design consistency and its potential impact on consumer behavior, several important gaps remain in the current literature.

First, existing studies have rarely examined visual design consistency as a system-level property of real commercial product ecosystems. Instead, many experimental investigations rely on simplified or artificially constructed stimuli, which may not fully capture the complexity of real-world product perception.

Second, the potential psychophysiological correlates of exposure to visually consistent versus less consistent product systems remain underexplored, particularly with respect to autonomic regulation as indexed by HRV and its relationship to behavioral and affective outcomes.

Third, there is limited understanding of how holistic visual coherence, as experienced in naturalistic commercial product environments, relates to concurrent changes in cognitive performance, autonomic activity, and self-reported emotional states.

To address these gaps, the present study adopts a within-subject design to examine whether exposure to commercial product systems characterized by differing levels of visual design consistency is associated with differences in brand recognition performance, HRV indices (SDNN and RMSSD), and state anxiety.

Importantly, this study focuses on ecologically valid commercial product systems in which multiple visual attributes naturally co-occur. Accordingly, the findings are interpreted as evidence of systematic associations between real-world product visual configurations and psychophysiological responses, rather than isolated effects of single visual features. Accordingly, the present study adopts a within-subject experimental design using real commercial product systems to examine behavioral, physiological, and self-reported responses under conditions of differing perceived visual design consistency.

## 2 Materials and methods

### 2.1 Participants

A total of 64 undergraduate students (35 males and 29 females) were initially recruited from Bengbu University through campus advertisements. All recruited participants received monetary compensation. After data collection, three participants were excluded due to incomplete or poor-quality physiological recordings following signal preprocessing and artifact rejection procedures for electrocardiography (ECG)-derived HRV data. The final valid sample consisted of 61 (34 males and 27 females) participants included in the statistical analyses.

Participants had a mean age of 19.1 years (SD = 1.19). All were native Chinese-speaking undergraduate students with normal or corrected-to-normal vision. None reported a history of cardiovascular, neurological, or psychiatric disorders. To minimize acute physiological confounds on HRV, participants were instructed to avoid caffeine intake, alcohol consumption, and vigorous physical activity for at least 12 h prior to the experiment (Laborde et al., 2017; Shaffer and Ginsberg, 2017).

To reduce expertise-related bias in visual perception and evaluation, all participants had no formal training or educational background in design-related disciplines (e.g., industrial design or visual communication design) (Parush et al., 2011). Participants were also naïve to the specific hypotheses of the study and had not previously participated in similar experiments.

No pre-experimental assessment of brand familiarity or brand attitude toward the stimulus brands was conducted prior to the experimental task. Participants were not informed of the identities of the target brands before the experiment.

No baseline state anxiety measurement was collected prior to the experimental task; the State-Trait Anxiety Inventory-State subscale (STAI-State) was administered only after each experimental condition to assess task-induced anxiety responses.

Sample size was determined based on empirical recommendations for within-subject behavioral and psychophysiological research and is consistent with prior HRV studies detecting medium-sized effects in cognitive-affective paradigms, rather than on a formal a priori power analysis (Brysbaert, 2019; Wickens and Keppel, 2004).

The study protocol was approved by the Bengbu Ethics Association (Approval No. 20240115) and was conducted in accordance with the Declaration of Helsinki. Written informed consent was obtained from all participants prior to the experiment, and participants were fully informed about the study objectives, procedures, potential risks, and their rights as research subjects.

HRV signals were visually inspected and preprocessed according to established guidelines for short-term HRV recording, including artifact correction and ectopic beat handling, ensuring data quality for subsequent analyses (Laborde et al., 2017; Task Force of the European Society of Cardiology and the North American Society of Pacing and Electrophysiology, 1996).

### 2.2 Experimental design and materials

#### 2.2.1 Experimental design

Rather than manipulating isolated visual variables experimentally, the present study employed real commercial product systems representing naturally occurring differences in overall perceived visual design consistency (Bloch, 1995; Crilly et al., 2004). This approach prioritized ecological validity while acknowledging reduced control over individual constituent visual dimensions (Mugge et al., 2015; Veryzer and Hutchinson, 1998).

A single-factor within-subjects design was implemented, with product visual design consistency (higher vs. lower perceived consistency) as the independent variable. Task order was counterbalanced across participants to control for potential order effects (Laborde et al., 2017; Wickens and Keppel, 2004). This counterbalancing procedure was also intended to distribute potential familiarity, practice, and fatigue effects associated with repeated exposure equally across the two experimental conditions, thereby reducing systematic bias attributable to task sequence.

The independent variable was operationalized as follows:

*Higher-consistency condition:* Product systems characterized by higher perceived visual design consistency, exhibiting stable and unified visual patterns across form language, color scheme, material expression, proportional structure, detail treatment, and stylistic identity (Mugge et al., 2015; Veryzer and Hutchinson, 1998).

*Lower-consistency condition*: Product systems characterized by lower perceived visual design consistency, exhibiting greater heterogeneity across the aforementioned visual dimensions (Bloch, 1995; Crilly et al., 2004).

The dependent variables were categorized into three domains:

*Physiological measures*: Time-domain indices of HRV, including standard deviation of normal-to-normal intervals (SDNN) and root mean square of successive differences (RMSSD), serving as objective indicators of autonomic nervous system activity (Laborde et al., 2017; Shaffer and Ginsberg, 2017).

*Subjective measures*: State-Trait Anxiety Inventory-State subscale (STAI-State) scores and perceived visual design consistency ratings, used to assess participants’ subjective emotional states and their perception of visual consistency (Crilly et al., 2004; Spielberger et al., 1983).

*Behavioral measures*: Accuracy in the brand identification task, defined as the proportion of stimuli correctly identified, reflecting behavioral-level processing performance (Desmet and Hekkert, 2007; Norman, 2004).

#### 2.2.2 Stimulus materials and standardization

Commercial products were selected to preserve ecological validity (Bloch, 1995; Crilly et al., 2004). Two commercial brands were selected to represent relatively high and relatively low levels of visual design consistency, according to the multidimensional definition introduced above (Mugge et al., 2015; Veryzer and Hutchinson, 1998). Visual design consistency was conceptualized and operationalized as a system-level perceptual property of product families rather than as an attribute of individual product features.

The selection of Xiaomi and Midea as operational exemplars was based on converging evidence from multiple independent sources:

First, established industrial design literature provides theoretical foundations for conceptualizing visual design consistency across product ecosystems. Research on product design strategy suggests that integrated brand ecosystems characterized by coherent visual language across product categories tend to exhibit higher perceived design consistency compared to function-oriented, category-dependent approaches (Karjalainen and Snelders, 2010; Mugge et al., 2015; Veryzer and Hutchinson, 1998).

Second, industry analyses and design case studies have documented systematic differences in design strategies across major consumer brands. Specifically, Xiaomi’s design approach has been characterized as emphasizing visual coherence and standardized patterns across product categories, reflecting a system-integrated strategy (Karjalainen and Snelders, 2010; Tong et al., 2021). In contrast, Midea’s design philosophy has been described as more function-driven and category-specific, resulting in greater visual heterogeneity across product lines (Qian et al., 2025).

Third, consumer research provides additional theoretical support for these classifications. Studies have demonstrated that brand design strategies emphasizing visual coherence across product lines are associated with higher processing fluency, brand recognition, and consumer preference (Orth and Malkewitz, 2008; Roster and Richins, 2009). Conversely, greater visual heterogeneity may influence cognitive processing and emotional responses (Poynor, 2003).

Fourth, preliminary perceptual evaluations and exploratory user interviews conducted prior to the experiment suggested converging patterns of perceived cross-category visual coherence, consistent with the literature-based classifications.

Finally, as supplementary validation, an independent study using identical stimulus materials with a separate sample of 60 participants (Yang and Wei, 2023) reported consistency ratings that aligned with the literature-based classifications (Xiaomi: mean = 1.52; Midea: mean = −0.30 on a 5-point semantic differential scale). While this prior study was conducted by the same research team, it employed independent participants and standardized assessment procedures, providing additional converging evidence for the stimulus classification.

These multiple converging sources of evidence—established design theory, industry documentation, consumer research, preliminary perceptual assessments, and independent validation—collectively informed the selection of stimulus brands. These preliminary assessments were used exclusively for stimulus selection and were not included in subsequent statistical analyses.

The stimulus set consisted of product images from six major small home appliance brands in the Chinese consumer market: Xiaomi, Midea, Supor, Joyoung, Gree, and Meiling. Xiaomi and Midea served as the target brands representing different levels of perceived system-level visual design consistency, while the remaining four brands served as filler stimuli. All products were selected from top-selling models in 2023 across six categories (rice cookers, electric pressure cookers, air fryers, portable blenders, electric kettles, and water purifiers), yielding a total of 36 images.

All images were standardized using Adobe Photoshop. Standardization procedures included:

- Removed all brand logos (Bloch, 1995; Veryzer and Hutchinson, 1998)
- Removed all text information on product images (Crilly et al., 2004)
- Unified image background to white (Holmqvist et al., 2011)
- Unified image resolution to 150 dpi (Wedel and Pieters, 2008)
- Unified image size to 600 × 600 pixels (Lavie, 2005)

Visual characteristics constituting product visual design consistency—including form language, color, material appearance, proportion, detail treatment, and overall stylistic coherence—were intentionally preserved. Therefore, low-level visual features were not experimentally controlled as independent variables (Bloch, 1995; Crilly et al., 2004).

**FIGURE 1:**
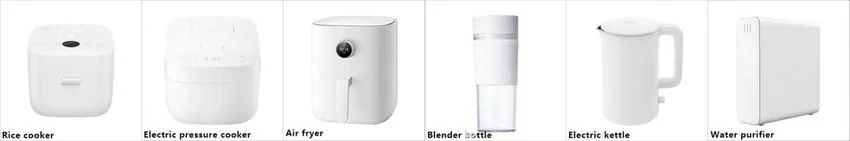
Tested small household appliances of the high visual-consistency brand (Xiaomi).

**FIGURE 2:**
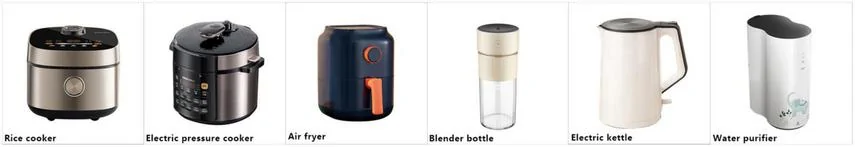
Tested small household appliances of the low visual-consistency brand (Midea).

The same filler stimuli were used in both experimental blocks to maintain task difficulty and ecological validity (Holmqvist et al., 2011; Wedel and Pieters, 2008). Examples of the experimental stimuli and the task paradigm are presented in Figures 1, 2.

#### 2.2.3 Measures

##### 2.2.3.1 State anxiety inventory

Participants’ momentary anxiety levels were assessed using the State Anxiety subscale of the Stateels were assessed ustory (STAI-State) (Spielberger et al., 1983). The Chinese version of the STAI-State has been culturally adapted and validated in Chinese populations (Zhang et al., 2015). The scale consists of 20 items rated on a 4-point Likert scale (1 = “not at all” to 4 = “very much so”), yielding a total score between 20 and 80, with higher scores indicating greater state anxiety. The STAI-State has demonstrated good reliability and validity in assessing task-induced transient anxiety (Spielberger et al., 1983).

##### 2.2.3.2 Perceived visual design consistency

Perceived visual design consistency was measured using a self-developed scale based on the Semantic Differential method (Osgood et al., 1957). The scale was developed through a Delphi expert consultation procedure involving experts in product design, psychology, and marketing (Boulkedid et al., 2011). The final scale comprises six dimensions (form, color, material and texture, symbols and patterns, craftsmanship, and usage method), with each dimension rated using a 5-point semantic differential format (−2 to +2). Higher total scores indicate higher perceived visual design consistency. The scale demonstrated good internal consistency (Cronbach’s α = 0.91) and a single-factor structure (cumulative variance explained = 71.45%, composite reliability = 0.925) in the present study.

##### 2.2.3.3 Heart rate variability (HRV)

HRV was recorded using a Healink-R211B portable electrocardiogram (ECG) recorder (Healink Ltd., China; bandwidth: 0.5–40 Hz; sampling rate: 400 Hz). HRV analysis focused on time-domain indices, including the standard deviation of normal-to-normal R deviation of norma and the root mean square of successive differences between adjacent RcR intervals (RMSSD), which are commonly used indicators of autonomic nervous system activity (Laborde et al., 2017; Shaffer and Ginsberg, 2017). All HRV data were processed using Kubios HRV Premium software (Version 3.1.0, Finland).

Respiratory activity was not externally controlled or monitored; participants were instructed to maintain natural breathing throughout the experiment. While RMSSD can be influenced by respiratory sinus arrhythmia, standardized breathing instructions, short task duration, and the within-subjects design were implemented to minimize potential respiratory confounds, consistent with established protocols in psychophysiological research (Laborde et al., 2017; Shaffer and Ginsberg, 2017).

### 2.3 Experimental procedures

The experiment was conducted in a sound-attenuated behavioral laboratory under constant lighting and controlled ambient conditions (temperature: 24 ± 1°C; relative humidity: 45 ± 5%) to minimize potential environmental influences on autonomic nervous system activity (Laborde et al., 2017; Shaffer and Ginsberg, 2017). Each participant completed the experiment individually in a seated position and was instructed to remain relaxed, avoid excessive body movements, and breathe naturally throughout the experiment (Laborde et al., 2017; Shaffer and Ginsberg, 2017).

A within-subject design was employed. Each participant completed two brand identification tasks corresponding to the high and low visual design consistency conditions. The same filler stimuli were presented in both task blocks. To minimize potential familiarity effects arising from repeated exposure, task order was counterbalanced across participants, thereby distributing any practice or repetition effects equally across experimental conditions (Laborde et al., 2017).

#### 2.3.1 Experimental interface

The experiment was conducted using a custom-designed computer program developed in Python and run on a Windows operating system. The program presented stimuli on a 6 × 5 grid layout, with each cell displaying one product image. Participants were instructed to identify and click on all products belonging to the target brand. Selected images were marked with a green dot to provide immediate visual feedback. Participants could select up to six images per trial. After completing their selections, participants clicked a “Submit” button to proceed to the next phase.

Upon submission, the program displayed the correct categorization of all 30 images, with products from the same brand arranged in rows (five rows total). This feedback allowed participants to verify their selections and understand the correct brand categorization. A “Restart” button was available to begin a new trial, which randomized the positions of all stimuli and cleared previous selections.

#### 2.3.2 Experimental procedure

The experimental procedure followed a standardized sequence for each participant:

*Step 1: resting baseline recording*

After providing written informed consent, participants were fitted with the electrocardiogram (ECG) recording device by the experimenter. Participants were then instructed to sit quietly for 3 min, during which resting-state HRV was recorded as a general physiological baseline (Laborde et al., 2017; Shaffer and Ginsberg, 2017). No additional baseline recording periods were obtained prior to each task condition.

This single-baseline approach is consistent with established protocols for within-subject HRV studies examining task-induced autonomic modulation, particularly when task order is counterbalanced and potential carryover effects are minimized through adequate rest intervals (Laborde et al., 2017; Shaffer and Ginsberg, 2017). The counterbalanced design helps to distribute any temporal effects evenly across conditions, reducing the impact of baseline drift on the comparison between conditions.

*Step 2: first task block*

Each participant completed the first brand identification task block corresponding to either the high visual design consistency condition (Xiaomi) or the low visual design consistency condition (Midea). The order of the two conditions was counterbalanced across participants.

For the first task block, the following sub-steps were executed:

*Step A: task instructions and exemplar presentation*

At the beginning of the task block, the system randomly selected one image from the six product images of the target brand and presented it to the participant as an exemplar, in order to clarify the target category and its visual design characteristics for the upcoming identification task.

*Step B: identification task execution*

**FIGURE 3:**
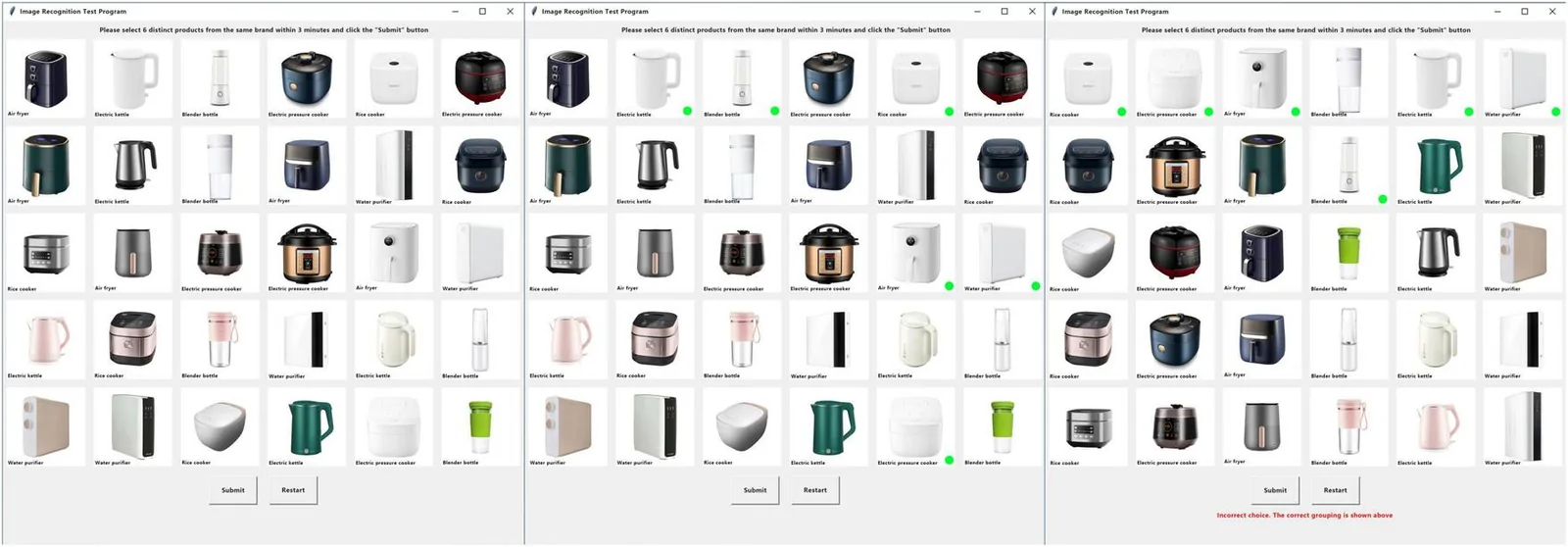
Schematic diagram of the recognition task interface procedure: (Left) Before recognition; (Middle) During recognition and selection; (Right) Program evaluation of the recognition results.

A total of 30 de-branded product images were displayed on the screen in a 6 × 5 grid layout, consisting of six products from the target brand (including the exemplar image) and 24 filler stimuli, with six products drawn from each of the remaining four brands. Stimulus positions were randomized on every trial. The interface procedure of the recognition task is illustrated in Figure 3.

Participants were instructed to identify and click on all products belonging to the target brand as quickly and accurately as possible within a 3-min time limit. The task was designed as a category-based visual identification task grounded in the perception of overall visual design language consistency, rather than a memory-matching task based on individual exemplars. Such tasks primarily rely on holistic perceptual processing of design features and visual coherence across product families (Desmet and Hekkert, 2007; Karjalainen and Snelders, 2010; Norman, 2004).

Throughout the task, ECG signals were continuously recorded. HRV indices were calculated based on the average of continuous 3-min ECG recordings during each task block, rather than on a trial-by-trial basis (Grégoire et al., 2023; Laborde et al., 2017; Shaffer and Ginsberg, 2017).

*Step C: post-task assessments*

Immediately after completing the identification task, participants completed the State Anxiety Inventory and the Visual Design Consistency Perception Scale on a computer (Laborde et al., 2017).

*Step D: rest period*

Participants then rested quietly for 2 min to allow autonomic activity to recover and to reduce potential carryover effects from the preceding task condition (Laborde et al., 2017).

*Step 3: second task block*

Following the rest period, participants proceeded to the second task block and repeated the same procedure (Steps AeD) until both brand conditions had been completed.

### 2.4 Data analysis

All statistical analyses were conducted using IBM SPSS Statistics version 26.0 (IBM Corp., Armonk, NY, United States), and HRV data preprocessing was performed using Kubios HRV Premium 3.1.0 (Tarvainen et al., 2014). The preprocessing procedures for HRV data were performed as described above. In accordance with established quality control standards for HRV analysis, datasets were excluded if the proportion of abnormal beats (e.g., artifacts or non-sinus beats) exceeded 5%, as such data are considered unreliable (Shaffer and Ginsberg, 2017; Task Force of the European Society of Cardiology and the North American Society of Pacing and Electrophysiology, 1996). After preprocessing, data from 61 of the 64 participants met the quality criteria and were retained for the final analyses (34 males and 27 females).

Prior to inferential analyses, the normality of paired difference scores between the high- and low-consistency conditions was assessed for all key dependent variables, including time-domain HRV indices, perceived visual design consistency ratings, the number of correctly identified brand products, and state anxiety scores, using the Shapiro–Wilk test (Shapiro and Wilk, 1965). The results indicated that the distributions of all paired differences significantly deviated from normality (all *p* < 0.001). Given the violation of the normality assumption for paired differences, nonparametric statistical methods were adopted to ensure robust inference. Specifically, Wilcoxon signed-rank tests were used to compare outcome measures between the high- and low-consistency conditions (Divine et al., 2013; Rosner et al., 2006).

All statistical tests were two-tailed, with the significance level set at α = 0.05. Unless otherwise specified, all statistical comparisons were conducted at the participant level using condition-level summary values. Descriptive statistics are reported as medians (*Mdn*) and interquartile ranges (*IQR*). Effect sizes were quantified using the *r* statistic, calculated as


$$\text{r}=\left|\text{Z}\right|/\sqrt{\text{N}}$$

where *Z* represents the standardized Wilcoxon test statistic and *N* denotes the number of valid paired observations (Cohen, 1992; Fritz et al., 2012).

All Wilcoxon signed-rank tests were computed using a unified contrast definition (high consistency minus low consistency), such that positive *Z*-values indicate higher values in the high-consistency condition.

According to Cohen’s (1992) guidelines, *r ≥* 0.50 was interpreted as a large effect, values between 0.30 and 0.49 as a medium effect, and values between 0.10 and 0.29 as a small effect.

Bias-corrected and accelerated (BCa) bootstrap confidence intervals were computed to provide robust interval estimates for the effect sizes. A total of 5,000 bootstrap resamples were generated with replacement from the original sample, and 95% confidence intervals were calculated using the bias-corrected and accelerated (BCa) method (Efron, 1979; Efron and Tibshirani, 1993). This nonparametric approach does not rely on distributional assumptions and provides robust interval estimates for the population parameters.

This analytical strategy is consistent with established recommendations for nonparametric analyses in psychophysiological research (Cumming, 2013; Laborde et al., 2017).

In addition, to rigorously evaluate potential confounds arising from low-level visual properties, we conducted comprehensive stimulus-level analyses using Python-based image processing tools (OpenCV, scikit-image). Specifically, we quantified mean luminance, visual entropy (Shannon entropy), and color distributions in the HSV space (Hue, Saturation, Value) for all experimental stimuli. These metrics were extracted to objectively characterize the perceptual differences between stimulus sets and to assess whether observed effects could be attributed to simple feature salience rather than design consistency *per se*.

## 3 Results

### 3.1 Manipulation check: post-task consistency ratings

To examine whether the manipulation of the independent variable—product visual design consistency (high vs. low)—was successful, a Wilcoxon signed-rank test was conducted to compare participants’ perceived visual design consistency ratings between the two conditions (Table 1).

**TABLE 1:** Comparison of subjective ratings on product visual design consistency (*N* = 61).

Measure	Paired median M (Q1, Q3)		*Z*	*p*	Effect size (r)
	High consistency (Xiaomi)	Low consistency (Midea)			
Consistency score	9.00 (7.0, 11.0)	2.00 (−2.0, 6.5)	5.91	< 0.001***	0.76

Values are expressed as medians with interquartile ranges (Q1, Q3). Subjective ratings of perceived product visual design consistency were compared between conditions using the Wilcoxon signed-rank test. Effect size: $r=\left|Z\right|/\sqrt{\text{N}}$.

***p < 0.001.

Descriptive statistics indicated that the median perceived consistency rating was substantially higher in the high-consistency condition than in the low-consistency condition (high consistency: *Mdn* = 9.00, *IQR* = 4.00; low consistency: *Mdn* = 2.00, *IQR* = 8.50). The Wilcoxon signed-rank test revealed a highly significant difference between the two conditions (*Z* = 5.91, *p* < 0.001), with a large effect size (*r* = 0.76). These findings confirm that participants perceived the two stimulus sets differently with respect to visual design consistency under the present experimental procedure.

To further evaluate the precision of the effect estimate, a bootstrap confidence interval for the median difference in perceived consistency ratings between the two conditions was computed based on 5,000 resamples. The results showed that the median difference in perceived consistency ratings (high consistency minus low consistency) was 7.00, with a 95% bootstrap confidence interval of [5.00, 7.00], providing additional evidence for a reliable difference in subjective consistency perception between the two conditions.

Because perceived consistency ratings were obtained after completion of the recognition task, these ratings should be interpreted as participants’ post-task evaluations rather than completely independent perceptual judgments.

**FIGURE 4:**
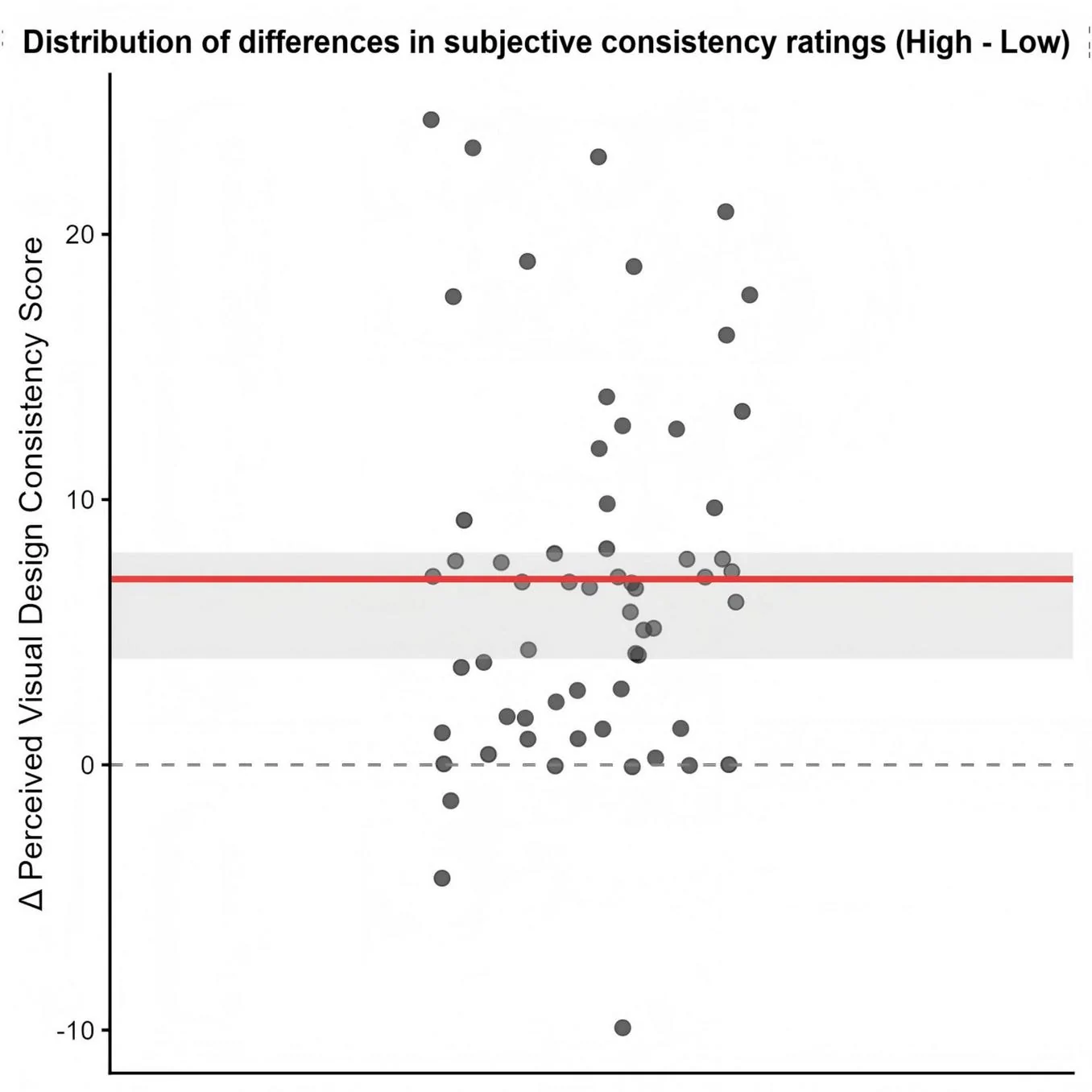
Distribution of differences in subjective consistency ratings (High consistency minus Low consistency) (*N* = 61). Each black point represents an individual participant’s difference score (jittered for visibility). The red horizontal line marks the median difference (ΔMdn = 7.00), with the gray band indicating the 95% confidence interval [95% CI (5.00, 7.00)] from 5,000 bootstrap samples. The distribution shows a clear skew toward positive values, confirming that Xiaomi products elicited significantly higher perceived consistency than Midea products.

For visual inspection of the distributional characteristics and the direction of the effect, Figure 4 presents the distribution of differences of perceived consistency ratings for both conditions.

### 3.2 Behavioral results: brand identification accuracy

To examine differences in brand identification performance between the high- and low-consistency conditions, the number of correctly identified products was analyzed. Because the paired differences across conditions did not meet the assumption of normality, a Wilcoxon signed-rank test was used to compare identification accuracy between conditions (Table 2).

**TABLE 2:** Comparison of accuracy in the brand identification task (*N* = 61).

Measure	Paired median M (Q1, Q3)		*Z*	*p*	Effect size (r)
	High consistency (Xiaomi)	Low consistency (Midea)			
Number of correct identifications	4.00 (3.50, 5.00)	2.00 (2.00, 3.00)	5.73	< 0.001***	0.73

Values are expressed as medians with interquartile ranges (Q1, Q3). Paired comparisons between high- and low-consistency conditions were conducted using the Wilcoxon signed-rank test. Effect size: $r=\left|Z\right|/\sqrt{\text{N}}$.

***p < 0.001.

Descriptive statistics showed that the median number of correct identifications was higher in the high-consistency condition (*Mdn* = 4.00, *IQR* = 1.50) than in the low-consistency condition (*Mdn* = 2.00, *IQR* = 1.00). The Wilcoxon signed-rank test revealed a highly significant difference between the two conditions (*Z* = 5.73, *p* < 0.001), with a large effect size (*r* = 0.73).

To further evaluate the precision of the estimated difference, a bootstrap confidence interval for the median difference in identification accuracy between the two conditions was computed based on 5,000 bootstrap resamples. The results showed that the median difference in the number of correct identifications (high-consistency minus low-consistency condition) was 2, with a 95% bootstrap confidence interval of [2, 3].

It should be noted that the stimulus systems may also differ in global visual complexity and color distribution patterns, which were not experimentally isolated from the manipulation of visual design consistency.

**FIGURE 5:**
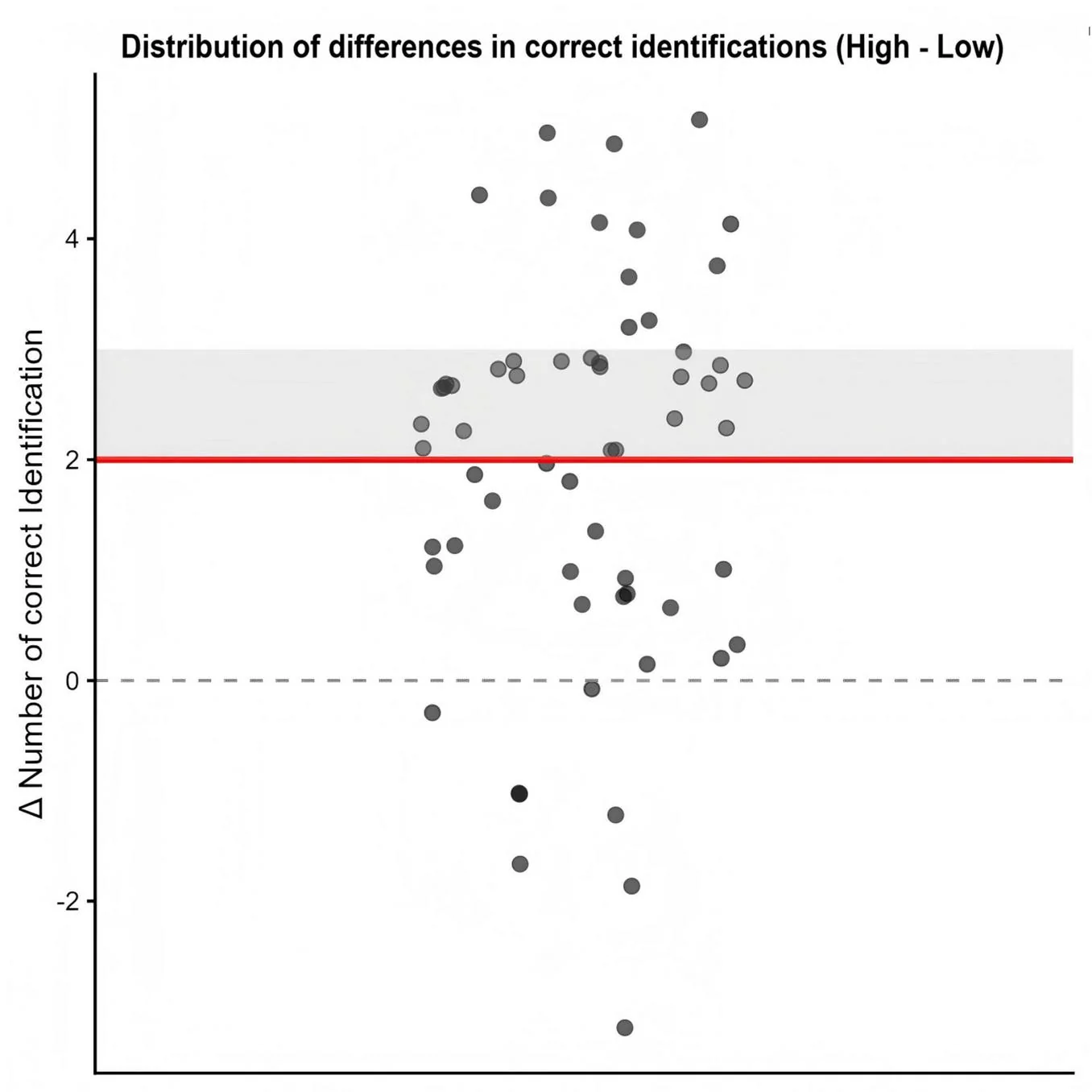
Distribution of differences in correct identifications (High consistency minus Low consistency) (*N* = 61). Black points represent individual accuracy differences. The red line marks the median improvement (ΔMdn = 2.00), with the 95% bootstrap confidence interval shown in gray. The data show that most participants performed better on the high-consistency (Xiaomi) task, though a small number exhibited reduced performance.

Figure 5 illustrates the distribution of individual differences in brand identification accuracy between the high- and low-consistency conditions.

### 3.3 Physiological response: task-evoked HRV changes

To examine differences in HRV between the high- and low-consistency conditions, HRV data collected during the brand identification task were analyzed. Because the paired differences of HRV indices between conditions did not satisfy the assumption of normality, Wilcoxon signed-rank tests were used to conduct paired comparisons (Table 3).

**TABLE 3:** Comparison of HRV metrics under different conditions (*N* = 61).

Measure	Paired median M (Q1, Q3)		*Z*	*p*	Effect size (r)
	High consistency (Xiaomi)	Low consistency (Midea)			
SDNN (ms)	46.54 (36.18, 60.76)	40.93 (32.72, 56.30)	2.41	0.016*	0.31
RMSSD (ms)	24.41 (19.57, 36.05)	23.19 (17.43, 32.59)	4.92	< 0.001***	0.61

Values represent task-phase HRV recorded during the brand identification task. Effect size: $r=\left|Z\right|/\sqrt{\text{N}}$. Baseline HRV values and baseline-adjusted analyses are reported in Supplementary Tables S1, S2.

*p < 0.05;

***p < 0.001.

The results revealed significant differences between the high- and low-consistency conditions for both time-domain HRV indices. Specifically, the median SDNN value was higher in the high-consistency condition (*Mdn* = 46.54 ms, *IQR* = 24.6) than in the low-consistency condition (*Mdn* = 40.93 ms, *IQR* = 23.6). The Wilcoxon signed-rank test yielded a statistically significant effect (*Z* = 2.41, *p* = 0.016), with a medium effect size (*r* = 0.31). A bootstrap analysis based on 5,000 resamples indicated that the median paired difference in SDNN was 2.64 ms, with a 95% confidence interval of [0.50, 4.74].

For RMSSD, the high-consistency condition also exhibited higher values (high consistency: *Mdn* = 24.41 ms, *IQR* = 16.4; low consistency: *Mdn* = 23.19 ms, *IQR* = 15.2). The Wilcoxon signed-rank test showed a highly significant difference between conditions (*Z* = 4.92, *p* < 0.001), accompanied by a large effect size (*r* = 0.61). The corresponding bootstrap analysis yielded a median paired difference of 2.10 ms, with a 95% confidence interval of [0.75, 3.24].

Both RMSSD and SDNN showed significant increases in the high-consistency condition, consistent with enhanced parasympathetic (vagal) modulation. Although RMSSD is widely regarded as a more specific index of vagally-mediated high-frequency heart rate variability, SDNN also substantially reflects parasympathetic activity, particularly in short-term (5-min) recordings where sympathetic influences on overall variability are limited (Laborde et al., 2017; Shaffer and Ginsberg, 2017). The convergence of both indices thus provides robust, multi-metric evidence that higher visual design consistency is associated with greater cardiac vagal tone, rather than isolated changes in a single autonomic subsystem (Inguscio et al., 2021; Rossi et al., 2017; Sarvi et al., 2026).

Baseline HRV indices did not differ significantly between conditions (all *p* > 0.05), indicating comparable resting autonomic states prior to task onset (see Supplementary Table S1).

Within-condition analyses further revealed that HRV indices were significantly reduced from baseline during the low-consistency condition, whereas no significant baseline-to-task differences were observed in the high-consistency condition (Supplementary Table S1).

Baseline-adjusted change score analyses (ΔHRV = task - baseline further confirmed this pattern, showing significantly smaller reductions in HRV in the high—consistency condition compared the low—consistency condition (Supplementary Table S2).

**FIGURE 6:**
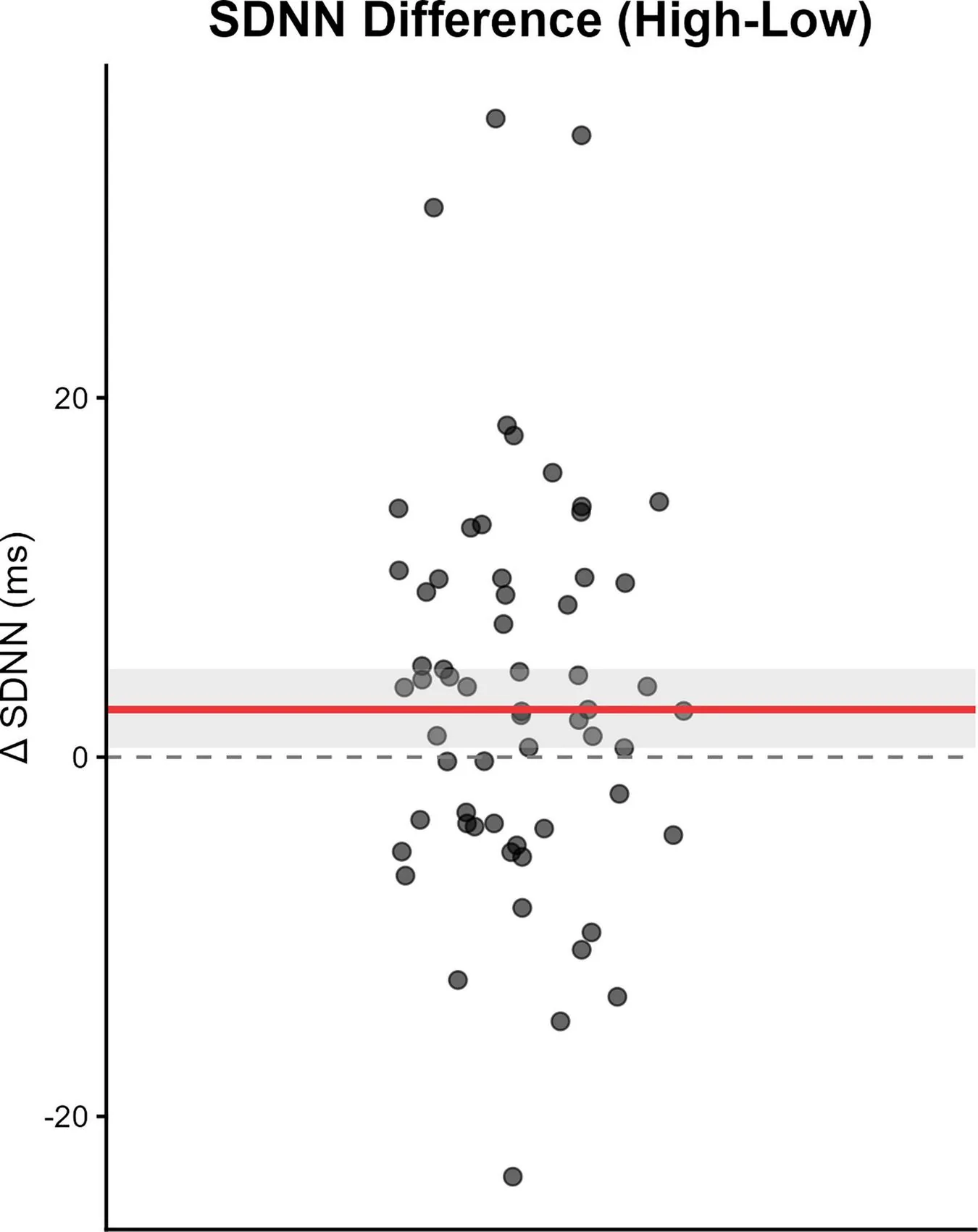
Distribution of differences in task-evoked SDNN (High consistency minus Low consistency) (*N* = 61). Individual ΔSDNN values are shown as jittered black points. The red line indicates the median paired difference (ΔMdn = 2.64 ms), with the 95% bootstrap confidence interval [95% CI (0.50, 4.74)] shown in gray. The positive shift suggests that the high-consistency task induced increased SDNN, reflecting enhanced autonomic flexibility.

**FIGURE 7:**
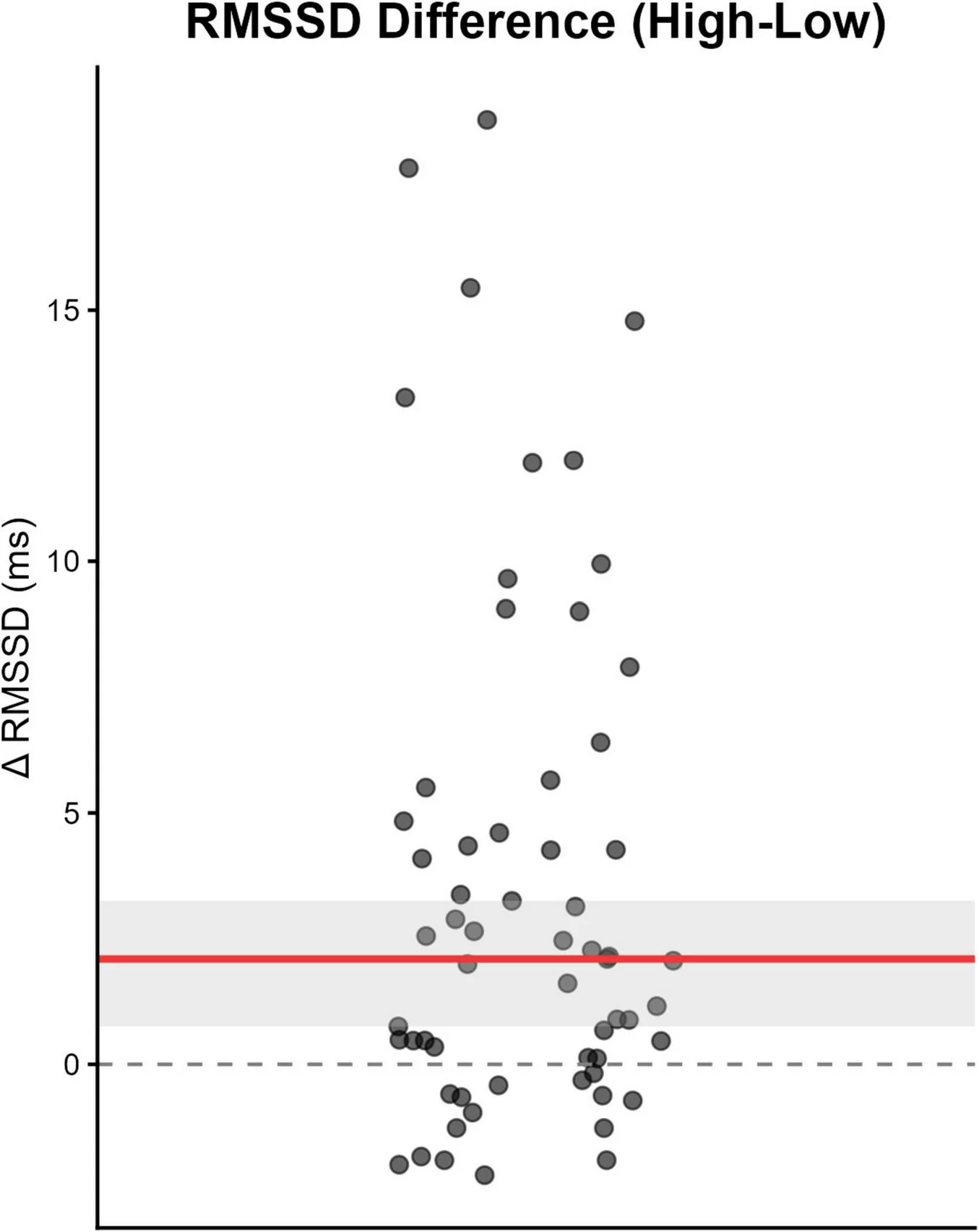
Distribution of differences in task-evoked RMSSD (High consistency minus Low consistency) (*N* = 61). ΔRMSSD values are shown as individual points. The red line marks the median paired difference (ΔMdn = 2.10 ms), with the 95% bootstrap confidence interval [95% CI (0.75, 3.24)] shown in gray. The right-skewed distribution confirms that high-consistency tasks generally elevated parasympathetic HRV, although a small number of participants showed decreases.

Figures 6, 7 illustrate the distributions of SDNN and RMSSD, respectively, under the two consistency conditions.

### 3.4 Subjective emotional state: post-task state anxiety

To examine differences in state anxiety between the high- and low-consistency conditions, state anxiety levels were assessed using the STAI-State after participants completed the brand identification task. As the paired differences in state anxiety scores between conditions did not meet the assumption of normality, Wilcoxon signed-rank tests were conducted for paired comparisons (Table 4).

**TABLE 4:** Comparison of state anxiety levels under different task conditions (*N* = 61).

Measure	Paired median M (Q1, Q3)		*Z*	*p*	Effect size (r)
	High consistency (Xiaomi)	Low consistency (Midea)			
State anxiety score	35.00 (30.00, 41.50)	38.00 (34.00, 44.30)	−3.08	0.002**	0.39

Values are expressed as medians with interquartile ranges (Q1, Q3). State anxiety levels were compared between conditions using the Wilcoxon signed-rank test. Effect size: $r=\left|Z\right|/\sqrt{\text{N}}$.

**p < 0.01.

Descriptive statistics indicated that the median STAI-State score following completion of the high-consistency condition was lower (*Mdn* = 35.00, *IQR* = 11.50) than that observed after the low-consistency condition (*Mdn* = 38.00, *IQR* = 10.30). The Wilcoxon signed-rank test revealed that this difference was statistically significant (*Z* = −3.08, *p* = 0.002), with an effect size of *r* = 0.39, corresponding to a medium effect.

To further assess the robustness of the effect estimate, a bootstrap confidence interval for the median paired difference was computed based on 5,000 resamples. The median difference in STAI-State scores (high-consistency minus low-consistency condition) was −2, with a 95% bootstrap confidence interval of [−4.00, 0].

**FIGURE 8:**
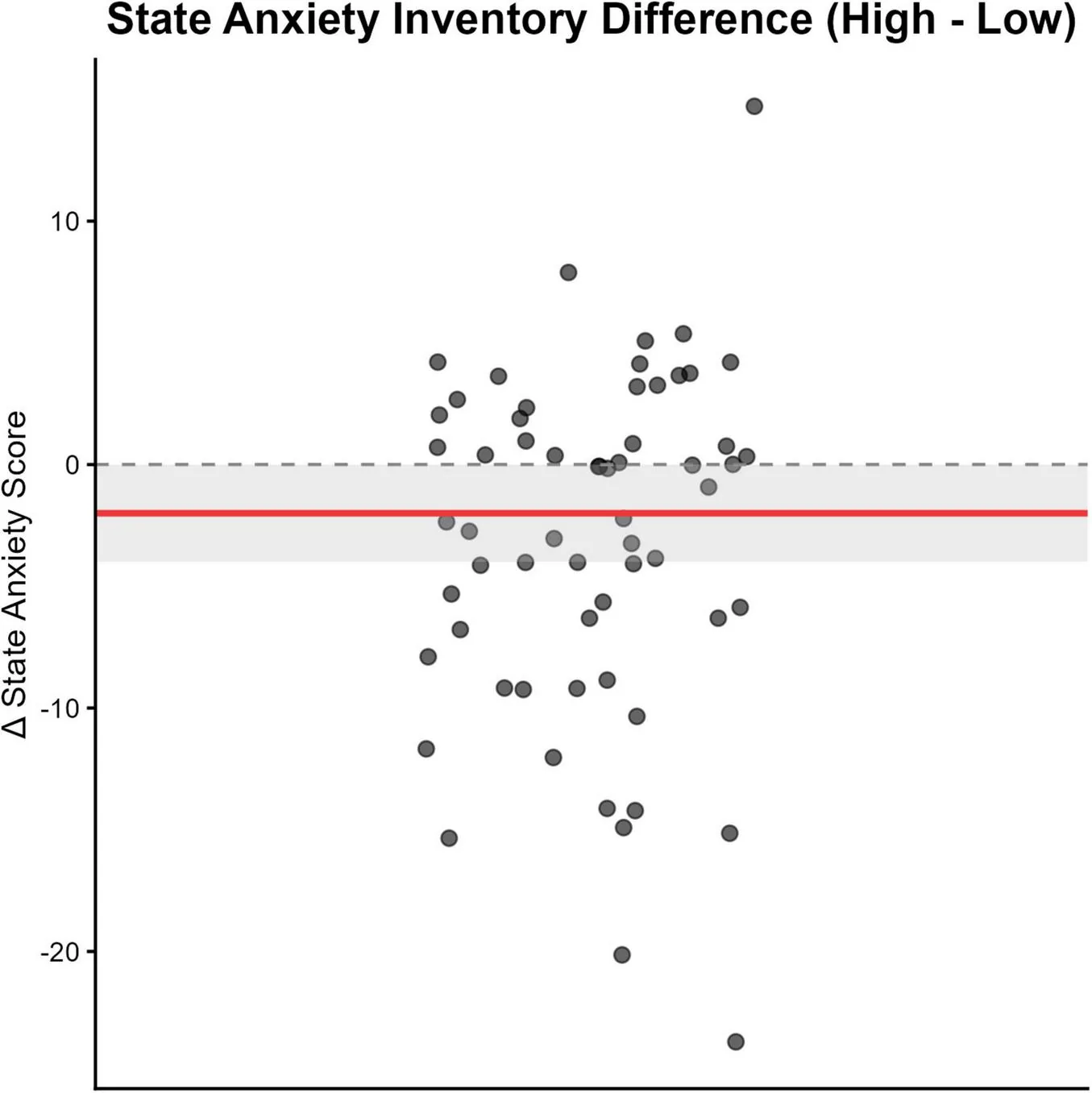
Distribution of differences in state anxiety scores (High consistency minus Low consistency) (*N* = 61). Each point represents an individual’s anxiety score difference (High-Low). The red line indicates the median difference (ΔMdn = −3.00), with the gray band showing the 95% confidence interval [95% CI (−5.00, −1.00)]. The distribution is strongly skewed toward negative values, indicating that the high-consistency (Xiaomi) task significantly reduced state anxiety relative to the low-consistency (Midea) task.

Figure 8 presents the distribution of differences illustrating the distribution of state anxiety scores across the two consistency conditions.

### 3.5 Supplementary analyses of low-level visual properties

To assess whether the observed behavioral and psychophysiological effects could be systematically attributed to low-level visual properties rather than overall visual design consistency, we conducted a series of supplementary stimulus-level analyses comparing the high-consistency (Xiaomi) and low-consistency (Midea) product image sets. Descriptive statistics for all experimental stimuli, including filler brands, are presented in Supplementary Table S3.

#### 3.5.1 Luminance and visual entropy

Mean luminance and visual entropy were extracted from each product image using Python-based image processing. Because only six product images were available in each experimental condition (*n* = 6), two-tailed Mann–Whitney U tests were adopted as the primary inferential analysis. This non-parametric approach is appropriate for small samples and is consistent with the analytical framework used for the participant-level experimental data.

The high-consistency stimulus set exhibited a higher mean luminance (*M* = 242.63, SD = 3.05) than the low-consistency set (*M* = 212.84, *SD* = 25.38). The Mann–Whitney analysis indicated a marginally significant difference with a large effect size (*U* = 30.00, *Z* = 1.92, *p* = 0.065, *r* = 0.56; Supplementary Table S4). In contrast, visual entropy did not differ significantly between conditions (*U* = 12.00, *Z* = −0.96, *p* = 0.394, *r* = 0.28; Supplementary Table S4).

As a robustness check, independent-samples *t*-tests were additionally performed. The results were broadly consistent with the primary analyses: luminance differed significantly between conditions with a large effect size, *t*(10) = 2.61, *p* = 0.047, *Cohen’s d* = 1.51 whereas visual entropy remained non-significant, *t*(10) = −1.03, *p* = 0.328, *Cohen’s d* = −0.59 (Supplementary Tables S5, S6). Taken together, these complementary analyses provide converging evidence for a luminance difference between the two stimulus sets while confirming the absence of reliable differences in visual entropy.

#### 3.5.2 HSV color distribution characteristics

Beyond scalar summary measures, normalized HSV color histograms were analyzed to characterize the chromatic composition of the experimental stimuli more comprehensively (Supplementary Table S7; see also Supplementary Figure S1).

Hue distributions showed that pixel values in the Xiaomi images were predominantly assigned to the H_0_ histogram bin, accounting for 89.25% of all pixels, reflecting the largely achromatic appearance of the stimulus set. No secondary hue peak exceeded 0.06%. By contrast, although the H_0_ bin remained dominant in the Midea images (71.22%), additional peaks were observed at H_2_ (red–orange, 8.21%) and H_19_ (blue, 4.30%), indicating a broader distribution of chromatic categories.

Saturation distributions further distinguished the two conditions. Xiaomi images were overwhelmingly concentrated in the lowest saturation bin (S_0_), with 98.05% of pixels exhibiting minimal saturation. In comparison, Midea images, while still predominantly low in saturation (74.13% at S_0_), contained a substantial proportion of pixels distributed across higher saturation bins (20.9% across S_1_–S_31_), consistent with the broader chromatic distribution described above.

Brightness (value) distributions revealed that both stimulus sets were generally composed of high-value pixels. However, Xiaomi images exhibited a more concentrated distribution in the highest brightness range, with 64.77% of pixels located in V_31_ and an additional 28.7% distributed across adjacent high-brightness bins (V_27_–V_30_). In contrast, although Midea images showed a comparable peak at V_31_ (66.39%), they displayed a broader value distribution extending into lower-brightness bins (V_0_–V_10_), consistent with the lower mean luminance observed in the preceding analyses.

Taken together, these supplementary analyses indicate that the two experimental stimulus sets differed in several low-level visual characteristics, particularly luminance and chromatic distribution, whereas no reliable difference in visual entropy was observed. These differences reflect inherent properties of the ecologically valid commercial product photographs used in the present study rather than experimentally manipulated stimulus attributes. Accordingly, these low-level visual characteristics should be considered when interpreting the present findings but are unlikely to fully account for the consistent behavioral, physiological, and subjective response patterns observed across multiple outcome measures (see section 4.2 for further discussion).

## 4 Discussion

The present exploratory study investigates the psychophysiological correlates associated with exposure to real commercial product systems characterized by different levels of perceived visual design consistency, rather than isolating the causal effects of any single visual design attribute. The convergence of behavioral, physiological, and subjective findings provides preliminary evidence that product systems characterized by higher perceived visual design consistency are associated with enhanced brand recognition performance, more favorable autonomic nervous system responses, and reduced post-task state anxiety. Below, we interpret these findings within the broader context of existing literature, address key methodological considerations, and outline implications for theory and practice.

### 4.1 Interpretation of the main findings

The present findings can be integrated within a multi-level theoretical framework that connects visual design features to cognitive, physiological, and emotional outcomes. At the perceptual level, processing fluency theory (Reber et al., 2004; Schwarz, 2004) suggests that visual consistency reduces the cognitive resources required for stimulus integration. The observed pattern of higher brand identification accuracy in the high-consistency condition is consistent with this theoretical perspective, indicating that more visually coherent product systems may facilitate faster and more accurate recognition.

At the physiological level, the neurovisceral integration model (Thayer and Lane, 2009) provides a framework for understanding how cognitive demands influence autonomic nervous system activity. The higher HRV values observed in the high-consistency condition—specifically increased SDNN and RMSSD—are consistent with reduced autonomic arousal and potentially enhanced emotional regulation capacity. These findings align with previous research demonstrating that lower cognitive load and more fluent processing are associated with higher parasympathetic activity (Laborde et al., 2017; Shaffer and Ginsberg, 2017).

At the emotional level, the cognitive-motivational-relational theory of emotion (Lazarus, 1991) suggests that reduced cognitive load may lead to more positive emotional states and lower anxiety. This interpretation is further supported by the broaden-and-build theory of positive emotions, which proposes that positive emotional states broaden individuals’ momentary thought–action repertoires and facilitate adaptive psychological and physiological functioning (Fredrickson, 2001). The lower STAI-State scores observed following exposure to the high-consistency condition are consistent with this theoretical framework, indicating that more visually coherent product systems may be associated with reduced subjective stress and more positive emotional experiences.

The convergence of findings across behavioral, physiological, and emotional domains provides compelling evidence for the multifaceted impact of visual design consistency. The improved brand identification accuracy (behavioral domain) suggests that consistent designs facilitate cognitive processing and recognition. The higher HRV values (physiological domain) indicate reduced autonomic arousal and potentially enhanced emotional regulation capacity. The lower state anxiety scores (emotional domain) reflect reduced subjective stress and more positive emotional experiences. This multi-method convergence strengthens the validity of our conclusions and demonstrates how different measurement approaches can provide complementary insights into user responses to design stimuli.

However, these associations should be interpreted within the broader context of real commercial product systems. The present study employed naturally occurring differences in overall perceived visual design consistency rather than experimentally isolating individual visual dimensions. Consequently, the observed effects cannot be attributed to any single constituent attribute in isolation.

### 4.2 Visual design consistency as a multidimensional construct

A key consideration in interpreting the present findings concerns the multidimensional nature of visual design consistency and its relationship to low-level visual properties such as color distribution, perceptual complexity, and feature-level organization. This has important implications for both the interpretation of the present results and the broader theoretical understanding of visual design consistency in industrial design contexts.

Within industrial design research, visual design consistency is conceptualized as an integrated perceptual construct rather than a collection of isolated visual variables (Homburg et al., 2017; Tuch et al., 2012). The construct encompasses coordinated coherence among multiple visual attributes—including form language, chromatic scheme, material expression, proportion, detail treatment, and overall stylistic coherence—that together shape consumers’ holistic perception of a product family (Desmet and Hekkert, 2007; Karjalainen and Snelders, 2010). From this perspective, chromatic coherence constitutes one integral dimension of visual design consistency rather than an independently manipulated experimental variable.

From a cognitive processing perspective, these multidimensional visual attributes may influence perception through mechanisms related to perceptual fluency, attentional allocation, and visual search efficiency. Prior research has demonstrated that visual complexity can modulate attentional resources and cognitive load during visual processing tasks (Donderi, 2006; Pieters et al., 2010; Rosenholtz et al., 2007). In particular, classical models of visual search distinguish between feature search, which is typically fast and parallel, and conjunction search, which requires more serial and effortful processing (Geisler and Perry, 2009; Treisman and Gelade, 1980). Within this framework, it is plausible that high-consistency stimuli facilitated more efficient feature-based processing, whereas low-consistency stimuli required more demanding integration of multiple visual features, thereby increasing perceptual and cognitive load.

Importantly, the present study was not designed to isolate the independent contributions of individual visual dimensions. Instead, we employed ecologically valid commercial product systems in which multiple visual attributes co-occur and jointly define perceived design consistency. This methodological choice prioritizes ecological validity and external realism, while necessarily limiting experimental control over individual low-level visual features. Consequently, the observed effects should be interpreted as arising from the integrated perceptual configuration of design consistency rather than from any single isolated visual attribute.

In addition to visual complexity, chromatic uniformity has been identified in prior research as a factor influencing perceptual fluency and attentional allocation in visual processing (Pieters et al., 2010; Reber et al., 2004; Rosenholtz et al., 2007). From a perceptual organization perspective, color regularity may facilitate perceptual grouping and reduce feature-level variability, thereby improving processing efficiency under certain visual conditions. In the present study, however, chromatic uniformity could not be independently manipulated or isolated from overall design consistency due to the use of real-world commercial stimuli. Accordingly, color-related influences are interpreted as embedded within the broader multidimensional construct of visual design consistency rather than as separable experimental factors.

Future research should therefore adopt experimental designs that allow for the independent manipulation of constituent visual dimensions. In particular, factorial designs (e.g., 2 × 2 manipulations of design consistency and chromatic uniformity) using systematically generated stimuli would enable more precise disentanglement of formal consistency effects and color-driven perceptual influences. Such approaches would complement the ecological validity of the present study by providing stronger causal inference regarding specific visual mechanisms.

### 4.3 Theoretical and practical contributions

The present study makes several contributions to the literature. First, at the methodological level, it demonstrates the utility of HRV as an objective physiological indicator of cognitive and emotional responses to product design stimuli, complementing traditional self-report measures. This methodological innovation bridges the gap between design research and psychophysiology, providing a more comprehensive understanding of user experiences with product designs.

Second, at the theoretical level, the study provides empirical evidence linking visual design consistency to both behavioral performance and autonomic nervous system activity. The convergence of findings across multiple measurement domains strengthens the theoretical foundation for understanding how visual design features influence user responses. These findings extend existing evidence by demonstrating that visual design consistency is associated with differences in processing fluency, autonomic regulation, and emotional experience.

Third, at the practical level, the study offers actionable insights for product designers seeking to optimize visual consistency for enhanced brand recognition and reduced user stress. The findings suggest that more visually coherent product systems may facilitate faster and more accurate brand identification while promoting more favorable physiological and emotional responses. However, these practical implications should be interpreted within the context of the present study’s exploratory and proof-of-concept nature.

Fourth, at the interdisciplinary level, the study exemplifies how neuroergonomic approaches can be integrated into consumer research to provide more comprehensive understanding of user experiences. The multimodal methodology combining behavioral, physiological, and self-report measures represents a promising direction for future research in product design and consumer neuroscience.

### 4.4 Study limitations and future research directions

Several limitations of the present study should be acknowledged, and future research may address these issues to provide more rigorous evidence regarding the psychophysiological effects of visual design consistency.

First, residual effects of brand familiarity and prior brand attitudes. Although explicit brand identifiers were removed, participants may still have recognized certain product systems based on accumulated marketplace experience. Brand familiarity has been shown to facilitate perceptual fluency and influence evaluation even in the absence of explicit brand cues (Janiszewski and Meyvis, 2001; Schwarz, 1990), while prior brand attitudes may also shape cognitive and affective processing (Percy and Rossiter, 1992). Recent neuromarketing studies further suggest that familiar brands can evoke measurable autonomic and neural responses even under implicit viewing conditions (Cartocci et al., 2021). Future studies should assess brand familiarity and pre-existing brand attitudes before the experiment so that these factors can be statistically controlled or examined as potential moderators.

Second, stimulus-level visual characteristics. Although supplementary stimulus-level analyses were conducted to quantify luminance, visual entropy, and HSV color distributions, the two product systems differed in several low-level visual characteristics, particularly luminance and chromatic uniformity. Because these low-level characteristics naturally covary with higher-order visual design consistency in real commercial product systems, they could not be experimentally disentangled in the present design. Consequently, the observed psychophysiological responses should be interpreted as reflecting the integrated influence of naturally co-occurring visual attributes rather than the isolated effect of any single visual property (Donderi, 2006; Pieters et al., 2010). Future studies employing factorial experimental designs with independently manipulated visual dimensions would allow stronger causal inference.

Third, manipulation check timing. Perceived visual design consistency was assessed after completion of the brand recognition task. Although post-task manipulation checks are standard practice in perceptual and aesthetic research (Isik and Vessel, 2021) and have been discussed extensively in experimental methodology (Alter and Oppenheimer, 2009), task engagement may have retrospectively biased consistency judgments. Future studies could obtain manipulation checks before the experimental task or collect trial-by-trial ratings to provide more direct evidence that perceived consistency is independent of task performance.

Fourth, HRV measurement considerations. Respiratory rate was not directly recorded, although participants were instructed to remain seated, breathe naturally, and minimize body movement throughout the experiment. Because respiratory sinus arrhythmia substantially influences RMSSD, future studies should include simultaneous respiratory monitoring to improve physiological interpretation of HRV indices (Laborde et al., 2017; Shaffer and Ginsberg, 2017).

Fifth, baseline HRV recording. A single resting baseline was obtained before both experimental blocks. Although the counterbalanced within-subject design helps distribute potential order effects across conditions, this approach cannot completely eliminate possible carry-over effects or autonomic adaptation between task blocks. Future studies could obtain separate baseline recordings before each condition and explicitly evaluate whether task order moderates HRV responses through sensitivity analyses.

Sixth, generalizability. Participants consisted exclusively of young Chinese university students, and the experimental stimuli represented only two brands within a single household appliance category. Replication across different cultural contexts, age groups, product categories, and brand ecosystems is therefore necessary before broader generalizations can be made.

Seventh, statistical power considerations. A *post-hoc* power analysis was conducted using G*Power 3.1 (Faul et al., 2007) for the Wilcoxon signed-rank test (matched pairs). Based on the observed effect sizes and a sample size of *N* = 61 (α = 0.05, two-tailed), the achieved power was 0.66 for SDNN (*r* = 0.31) and 0.85 for state anxiety (*r* = 0.39). While the power for SDNN falls slightly below the conventional threshold of 0.80 (Cohen, 1988), indicating moderate power for detecting this medium-sized effect, the overall reliability of our conclusions is supported by the convergence of evidence across multiple domains. Specifically, the RMSSD index exhibited a large effect size (*r* = 0.61), yielding a *post-hoc* power exceeding 0.99, and behavioral measures (e.g., brand identification accuracy) also demonstrated highly significant differences between conditions. Therefore, while we acknowledge the moderate power for SDNN, the multi-method convergence across physiological (RMSSD), subjective (STAI-State), and behavioral outcomes provides robust support for our conclusions. Future research should employ formal a priori power analyses to determine optimal sample sizes for detecting medium-sized physiological effects.

Eighth, state anxiety assessment. State anxiety was measured only after completion of the experimental tasks. Without a pre-task baseline assessment, changes attributable specifically to the experimental manipulation cannot be quantified directly. Future studies should incorporate both baseline and post-task State Anxiety Inventory assessments to evaluate changes in anxiety more precisely.

The following research agenda directly addresses the limitations outlined above while extending the present findings toward a broader understanding of visual design consistency and its psychophysiological consequences.

Future research should focus on several complementary directions.

First, individual difference moderators should be examined, including design expertise, cognitive style, aesthetic sensitivity, and cultural background, to determine whether these factors influence responses to visual design consistency.

Second, dimensional decomposition of visual design consistency deserves particular attention. Factorial experiments independently manipulating constituent visual dimensions—including form language, chromatic uniformity, material expression, and proportional relationships—would clarify the respective contributions of individual design attributes while strengthening causal inference.

Third, experimentally generated stimuli may complement studies using real commercial products. Although such stimuli inevitably sacrifice some ecological validity, they would permit systematic manipulation of isolated visual variables under tightly controlled experimental conditions.

Fourth, cross-cultural validation should investigate whether the psychophysiological effects of visual design consistency generalize across different cultural environments and consumer populations.

Finally, longitudinal and real-world investigations are needed to determine whether the short-term psychophysiological responses observed in laboratory settings translate into enduring effects on brand preference, consumer decision making, and long-term brand loyalty.

## 5 Conclusion

Using a within-subject psychophysiological design integrating behavioral performance, HRV, and self-reported anxiety, the present exploratory study found that participants exposed to real product systems characterized by higher perceived visual design consistency consistently exhibited patterns of response including higher brand recognition performance, higher HRV indices, and lower post-task anxiety.

Taken together, these convergent findings provide evidence of systematic associations consistent with the possibility that more visually coherent product systems are associated with more fluent cognitive processing and more adaptive affective and autonomic responses. Importantly, these findings should be interpreted as correlational rather than causal, given the use of naturally occurring commercial product systems.

More broadly, this exploratory study extends consumer neuroscience into product design research by demonstrating the feasibility of integrating psychophysiological measures with traditional behavioral and subjective assessments when investigating consumers’ responses to ecologically valid product systems.

At the same time, as an exploratory investigation, because the present study employed real commercial brands and manipulated a multidimensional design construct encompassing form, color, material appearance, proportion, detail treatment, and overall stylistic coherence, the observed multimodal associations should be interpreted as correlational evidence rather than causal effects. Future studies employing independently manipulated visual dimensions, experimentally generated stimuli, and more diverse participant samples will be essential for clarifying the relative contributions of specific visual characteristics and extending the generalizability of the present findings.

## Data Availability

The raw data supporting the conclusions of this article will be made available by the authors, without undue reservation.

## References

[B1] Aaker D. A. Keller K. L. (1990). Consumer evaluations of brand extensions. *J. Market.* 54 27–41. 10.2307/1252171

[B2] Alter A. L. Oppenheimer D. M. (2009). Suppressing secrecy through metacognitive ease: cognitive fluency encourages self-disclosure. *Psychol. Sci.* 20 1414–1420. 10.1111/j.1467-9280.2009.02461.x 19845889

[B3] Bloch P. H. (1995). Seeking the ideal form: product design and consumer response. *J. Market.* 59 16–29. 10.1177/002224299505900302

[B4] Boulkedid R. Abdoul H. Loustau M. Sibony O. Alberti C. (2011). Using and reporting the delphi method for selecting healthcare quality indicators: a systematic review. *PLoS One* 6:e20476. 10.1371/journal.pone.0020476 21694759 PMC3111406

[B5] Brysbaert M. (2019). How many participants do we have to include in properly powered experiments? A tutorial of power analysis with reference tables. *J. Cogn.* 2 1–38. 10.5334/joc.72 31517234 PMC6640316

[B6] Cartocci G. Rossi D. Modica E. Maglione A. G. Martinez Levy A. C. Cherubino P. et al. (2021). NeuroDante: poetry mentally engages more experts but moves more non-experts, and for both the cerebral approach tendency goes hand in hand with the cerebral effort. *Brain Sci.* 11:281. 10.3390/brainsci11030281 33668815 PMC7996310

[B7] Cohen J. (1988). *Statistical Power Analysis for the Behavioral Sciences*, 2nd Edn. Mahwah, NJ: Lawrence Erlbaum Associates. 10.4324/9780203771587

[B8] Cohen J. (1992). A power primer. *Psychol. Bull.* 112 155–159. 10.1037/0033-2909.112.1.155 19565683

[B9] Crilly N. Moultrie J. Clarkson P. J. (2004). Seeing things: consumer response to the visual domain in product design. *Design Stud.* 25 547–577. 10.1016/j.destud.2004.03.001

[B10] Cumming G. (2013). The new statistics: why and how. *Psychol. Sci.* 25 7–29. 10.1177/0956797613504966 24220629

[B11] Desmet P. Hekkert P. (2007). Framework of product experience. *Int. J. Design* 1 13–23.

[B12] Divine G. Norton H. J. Hunt R. Dienemann J. (2013). A review of analysis and sample size calculation considerations for wilcoxon tests. *Anesth. Anal.* 117 699–710. 10.1213/ANE.0b013e31827f53d7 23456667

[B13] Donderi D. C. (2006). Visual complexity: a review. *Psychol. Bull.* 132 73–97. 10.1037/0033-2909.132.1.73 16435958

[B14] Efron B. (1979). Computers and the theory of statistics: thinking the unthinkable. *SIAM Rev.* 21 460–480. 10.1137/1021092

[B15] Efron B. Tibshirani R. J. (1993). *An Introduction to the Bootstrap.* New York, NY: Chapman and Hall/CRC.

[B16] Faul F. Erdfelder E. Lang A.-G. Buchner A. (2007). G*Power 3: a flexible statistical power analysis program for the social, behavioral, and biomedical sciences. *Behav. Res. Methods* 39 175–191. 10.3758/BF03193146 17695343

[B17] Fredrickson B. L. (2001). The role of positive emotions in positive psychology: the broaden-and-build theory of positive emotions. *Am. Psychol.* 56 218–226. 10.1037/0003-066X.56.3.218 11315248 PMC3122271

[B18] Fritz C. O. Morris P. E. Richler J. J. (2012). Effect size estimates: current use, calculations, and interpretation. *J. Exp. Psychol.* 141 2–18. 10.1037/a0024338 21823805

[B19] Geisler W. S. Perry J. S. (2009). Contour statistics in natural images: grouping across occlusions. *Vis. Neurosci.* 26 109–121. 10.1017/S0952523808080875 19216819 PMC2660385

[B20] Grégoire J.-M. Gilon C. Carlier S. Bersini H. (2023). Autonomic nervous system assessment using heart rate variability. *Acta Cardiol.* 78 648–662. 10.1080/00015385.2023.2177371 36803313

[B21] Holmqvist K. Nyström M. Andersson R. Dewhurst R. Jarodzka H. van de Weijer J. (2011). *Eye Tracking: A Comprehensive Guide to Methods and Measures.* Oxford: Oxford University Press.

[B22] Homburg C. Jozić D. Kuehnl C. (2017). Customer experience management: toward implementing an evolving marketing concept. *J. Acad. Market. Sci.* 45 377–401. 10.1007/s11747-015-0460-7

[B23] Inguscio B. M. Cartocci G. Modica E. Rossi D. Martinez-Levy A. C. Cherubino P. et al. (2021). Smoke signals: a study of the neurophysiological reaction of smokers and non-smokers to smoking cues inserted into antismoking public service announcements. *Int. J. Psychophysiol.* 167 22–29. 10.1016/j.ijpsycho.2021.06.010 34175349

[B24] Isik A. I. Vessel E. A. (2021). From visual perception to aesthetic appeal: brain responses to aesthetically appealing natural landscape movies. *Front. Hum. Neurosci.* 15:676032. 10.3389/fnhum.2021.676032 34366810 PMC8336692

[B25] Janiszewski C. Meyvis T. (2001). Effects of brand logo complexity, repetition, and spacing on processing fluency and judgment. *J. Consum. Res.* 28 18–32. 10.1086/321945

[B26] Karjalainen T.-M. Snelders D. (2010). Designing visual recognition for the brand. *J. Prod. Innovat. Manage.* 27 6–22. 10.1111/j.1540-5885.2009.00696.x

[B27] Laborde S. Mosley E. Thayer J. F. (2017). Heart rate variability and cardiac vagal tone in psychophysiological research–recommendations for experiment planning, data analysis, and data reporting. *Front. Psychol.* 8:213. 10.3389/fpsyg.2017.00213 28265249 PMC5316555

[B28] Lavie N. (2005). Distracted and confused?: selective attention under load. *Trends Cogn. Sci.* 9 75–82. 10.1016/j.tics.2004.12.004 15668100

[B29] Lazarus R. S. (1991). Progress on a cognitive-motivational-relational theory of emotion. *Am. Psychol.* 46 819–834. 10.1037/0003-066X.46.8.819 1928936

[B30] Mugge R. Schoormans J. P. L. Schifferstein H. N. J. (2015). Design strategies to postpone consumers’ product replacement: the value of a strong person-product relationship. *Design J.* 8 38–48. 10.2752/146069205789331637

[B31] Norman D. A. (2004). *Emotion Design: Why We Love (or hate) Everyday Things.* New York, NY: Basic Books.

[B32] Orth U. R. Malkewitz K. (2008). Holistic package design and consumer brand impressions. *J. Market.* 72 64–81. 10.1509/JMKG.72.3.064

[B33] Osgood C. E. Suci G. J. Tannenbaum P. H. (1957). *The Measurement of Meaning.* Champaign, IL: University of Illinois Press.

[B34] Park C. W. Milberg S. Lawson R. (1991). Evaluation of brand extensions: the role of product feature similarity and brand concept consistency. *J. Consum. Res.* 18 185–193. 10.1086/209251

[B35] Parush A. Gauthier M. S. Arseneau L. Tang D. (2011). The human factors of night vision goggles: perceptual, cognitive, and physical factors. *Rev. Hum. Fact. Ergon.* 7 238–279. 10.1177/1557234X11410392

[B36] Percy L. Rossiter J. R. (1992). A model of brand awareness and brand attitude advertising strategies. *Psychol. Market.* 9 263–274. 10.1002/mar.4220090402

[B37] Pieters R. Wedel M. Batra R. (2010). The stopping power of advertising: measures and effects of visual complexity. *J. Market.* 74 48–60. 10.1509/jmkg.74.5.48

[B38] Plassmann H. Venkatraman V. Huettel S. Yoon C. (2015). Consumer neuroscience: applications, challenges, and possible solutions. *J. Market. Res.* 52 427–435. 10.1509/jmr.14.0048

[B39] Poynor R. (2003). *No More Rules: Graphic Design and Postmodernism.* London: Laurence King Publishing.

[B40] Qian X. Wang Y. Zhang L. (2025). Research on the development of product gene theory from the perspective of tangible and intangible dimensions [实体与非实体视阈的产品基因理论发展研究]. *Furniture Interior Decorat. (Jiaju Yu Shinei Zhuangshi)* 32 52–59. 10.16771/j.cn43-1247/ts.2025.11.009

[B41] Reber R. Schwarz N. Winkielman P. (2004). Processing fluency and aesthetic pleasure: Is beauty in the perceiver’s processing experience? *Pers. Soc. Psychol. Rev.* 8 364–382. 10.1207/s15327957pspr0804_3 15582859

[B42] Reimann M. Zaichkowsky J. Neuhaus C. Bender T. Weber B. (2010). Aesthetic package design: a behavioral, neural, and psychological investigation. *J. Consum. Psychol.* 20 431–441. 10.1016/j.jcps.2010.06.009

[B43] Rosenholtz R. Li Y. Nakano L. (2007). Measuring visual clutter. *J. Vis.* 7 17–17. 10.1167/7.2.17 18217832

[B44] Rosner B. Glynn R. J. Lee M.-L. T. (2006). The wilcoxon signed rank test for paired comparisons of clustered data. *Biometrics* 62 185–192. 10.1111/j.1541-0420.2005.00389.x 16542245

[B45] Rossi D. Maglione A. G. Modica E. Di Flumeri G. Venuti I. Brizi A. et al. (2017). “An eye tracking index for the salience estimation in visual stimuli,” in *Proceedings of the 2017 39th Annual International Conference of the IEEE Engineering in Medicine and Biology Society (EMBC)*, (Jeju). 10.1109/EMBC.2017.8037852 29060893

[B46] Roster C. A. Richins M. L. (2009). Ambivalence and attitudes in consumer replacement decisions. *J. Consum. Psychol.* 19 48–61. 10.1016/j.jcps.2008.12.008

[B47] Sarvi F. Aliannejadi M. Schelter S. Rijke M. D. (2026). Understanding visual saliency of outlier items in product search. *ACM Trans. Informat. Syst.* 44 1–29. 10.1145/3806228

[B48] Schwarz N. (1990). “Feelings as information: informational and motivational functions of affective states,” in *Handbook of Motivation and Cognition: Foundations of Social Behavior*, eds Higgins E. T. Sorrentino R. M. (Cham: Springer), 527–561.

[B49] Schwarz N. (2004). Metacognitive experiences in consumer judgment and decision making. *J. Consum. Psychol.* 14 332–348. 10.1207/S15327663JCP1404_2

[B50] Shaffer F. Ginsberg J. P. (2017). An overview of heart rate variability metrics and norms. *Front. Public Health* 5:258. 10.3389/fpubh.2017.00258 29034226 PMC5624990

[B51] Shapiro S. S. Wilk M. B. (1965). An analysis of variance test for normality (complete samples). *Biometrika* 52 591–611. 10.1093/biomet/52.3-4.591

[B52] Spielberger C. D. Gorsuch R. L. Lushene R. E. Vagg P. R. Jacobs G. A. (1983). *Manual for the State-Trait Anxiety Inventory (Form Y1 – Y2).* Sunnyvale, CA: Consulting Psychologists Press.

[B53] Tarvainen M. P. Niskanen J. P. Lipponen J. A. Ranta-Aho P. O. Karjalainen P. A. (2014). Kubios HRV–heart rate variability analysis software. *Comput. Methods Prog. Biomed.* 113 210–220. 10.1016/j.cmpb.2013.07.024 24054542

[B54] Task Force of the European Society of Cardiology and the North American Society of Pacing and Electrophysiology (1996). Heart rate variability: standards of measurement, physiological interpretation, and clinical use. *Circulation* 93 1043–1065. 10.1161/01.CIR.93.5.1043 8598068

[B55] Thayer J. F. Lane R. D. (2009). Claude Bernard and the heart–brain connection: further elaboration of a model of neurovisceral integration. *Neurosci. Biobehav. Rev.* 33 81–88. 10.1016/j.neubiorev.2008.08.004 18771686

[B56] Tong T. W. Guo Y. Chen L. (2021). How Xiaomi redefined what it means to be a platform. *Harvard Bus. Rev.* 1–4. Available online at: https://hbr.org/2021/09/how-xiaomi-redefined-what-it-means-to-be-a-platform

[B57] Treisman A. M. Gelade G. (1980). A feature-integration theory of attention. *Cogn. Psychol.* 12 97–136. 10.1016/0010-0285(80)90005-5 7351125

[B58] Tuch A. N. Presslaber E. E. Stöcklin M. Opwis K. Bargas-Avila J. A. (2012). The role of visual complexity and prototypicality regarding first impression of websites: working towards understanding aesthetic judgments. *Int. J. Hum. Comput. Stud.* 70 794–811. 10.1016/j.ijhcs.2012.06.003

[B59] Vecchiato G. Maglione A. G. Cherubino P. Wasikowska B. Wawrzyniak A. Latuszynska A. et al. (2014). Neurophysiological tools to investigate consumer’s gender differences during the observation of TV commercials. *Comput. Math. Methods Med.* 2014:912981. 10.1155/2014/912981 25147579 PMC4134790

[B60] Veryzer R. W. Jr. Hutchinson J. W. (1998). The influence of unity and prototypicality on aesthetic responses to new product designs. *J. Consum. Res.* 24 374–394. 10.1086/209516

[B61] Wedel M. Pieters R. (2008). Foundations and trends in marketing. *Found. Trends§Market.* 1 231–320. 10.1561/1700000011

[B62] Wickens T. D. Keppel G. (2004). *Design and Analysis: A Researcher’s Handbook.* Hoboken, NJ: Pearson Prentice-Hall.

[B63] Yang Y. Wei Y. (2023). Consistency of small household appliance product design language based on fuzzy cognition experiment [基于模糊认知实验的小家电产品设计语言一致性研究]. *J. Anyang Inst. Technol. (Anyang Gongxueyuan Xuebao)* 22 52–59. 10.19329/j.cnki.1673-2928.2023.02.010

[B64] Zhang Y. Li R. L. G. Mao S. Yuan Y. (2015). The reliability and validity of a Chinese-version short health anxiety inventory: an investigation of university students. *Neuropsychiatr. Dis. Treat.* 11 1739–1747. 10.2147/NDT.S83501 26213472 PMC4509540

