# Supplementary material for: Effects of product visual design consistency on brand recognition and state anxiety: a psychophysiological study integrating heart rate variability and self-report measures
Source: Front Psychol. 2026 Aug 11;17:1841820. doi: 10.3389/fpsyg.2026.1841820 (PMC13503611; doi:10.3389/fpsyg.2026.1841820)
Supplement: Supplementary file 1 [file Supplementary_file_1.docx]

# ****Supplementary Tables S1-S7****

****Supplementary Table S1.**** Within-condition comparisons of HRV indices between baseline and task phases (N=61)

| **Condition** | **HRV index** | **Baseline Mdn (IQR)** | **Task Mdn (IQR)** | ***Z*** | **p** | **Effect size (r)** |
| --- | --- | --- | --- | --- | --- | --- |
| High consistency | SDNN (ms) | 49.66 (33.34,61.53) | 46.54 (36.18, 60.76) | 0.54 | 0.593 | 0.07 |
| High consistency | RMSSD (ms) | 26.53 (18.32 , 37.87) | 24.41 (19.57, 36.05) | 0.73 | 0.466 | 0.09 |
| Low consistency | SDNN (ms) | 49.66 (33.34,61.53) | 40.93 (32.72, 56.30) | 2.34 | 0.019* | 0.30 |
| Low consistency | RMSSD (ms) | 26.53 (18.32 , 37.87) | 23.20 (17.43, 32.59) | 3.34 | 0.001*** | 0.43 |
| *Note:* Baseline HRV values were identical across conditions, as resting recordings were obtained once prior to task blocks. Wilcoxon signed-rank tests were used for all within-condition comparisons.  Effect size: $\text{r=\vert}\text{Z}\text{\vert/}\sqrt{\text{N}}$ | | | | | | |
| **p*<0.05;***p*<0.01;****p*<0.001 | | | | | | |

****Supplementary Table S2.**** Baseline-adjusted HRV change scores (ΔHRV = task − baseline) across conditions (N=61)

| **HRV index** | **Paired Median M (Q1, Q3)** | | ***Z*** | ***p*** | **Effect size (r)** |
| --- | --- | --- | --- | --- | --- |
|  | **High consistency ΔHRV Mdn (IQR)** | **Low consistency ΔHRV Mdn (IQR)** |  |  |  |
| ΔSDNN (ms) | 1.90(-6.58,7.83) | -2.98(-11.65,5.14) | 2.41 | 0.016** | 0.31 |
| ΔRMSSD (ms) | 0.83(-2.97,4.15) | -2.73(-5.43,1.51) | 4.92 | <0.001*** | 0.63 |
| *Note:* Positive values indicate increases relative to baseline. Statistical comparisons were conducted using Wilcoxon signed-rank tests.  Effect size: $\text{r }\text{= \vert}\text{Z}\text{\vert/}\sqrt{\text{N}}$ | | | | | |
| **p*<0.05;***p*<0.01;****p*<0.001 | | | | | |

****Supplementary Table S3.**** Table Descriptive Statistics of Low-Level Visual Properties Across All Brands

| **Brand** | **Condition** | **N** | **Mean Luminance** | **SD** | **Mean Entropy** | **SD** |
| --- | --- | --- | --- | --- | --- | --- |
| Xiaomi | High Consistency | 6 | 242.63 | 3.05 | 3.146 | 0.511 |
| Midea | Low Consistency | 6 | 212.84 | 25.38 | 3.542 | 0.692 |
| Joyoung | Filler | 6 | 226.78 | 14.63 | 3.004 | 0.440 |
| Meiling | Filler | 6 | 201.66 | 24.87 | 3.490 | 0.915 |
| Supor | Filler | 6 | 218.14 | 24.21 | 3.246 | 0.850 |
| Gree | Filler | 6 | 212.52 | 22.61 | 3.131 | 0.570 |
| *Note*: This table presents descriptive statistics of low-level visual properties across all stimuli. Filler brands were included for stimulus control purposes and were not part of the primary inferential analyses. | | | | | | |

****Supplementary Table S4.**** Primary Analysis - Mann‑Whitney U Test Results

| **Visual Property** | **Condition** | | ***U*** | ***Z*** | ***p*** | **Effect size (r)** |
| --- | --- | --- | --- | --- | --- | --- |
|  | **High consistency (Xiaomi)** | **Low consistency (Midea)** |  |  |  |  |
| Mean Luminance | 242.63 | 212.84 | 30.00 | 1.92 | 0.065 | 0.56 |
| Mean Entropy | 3.146 | 3.542 | 12.00 | -0.96 | 0.394 | 0.28 |
| *Note*: Mann–Whitney U tests (two-tailed) were used to compare low-level visual properties between conditions.  Effect size: $\text{r }\text{= \vert}\text{Z}\text{\vert/}\sqrt{\text{N}}$ | | | | | | |
| **p*<0.05;***p*<0.01;****p*<0.001 | | | | | | |

****Supplementary Table S5.**** Robustness Check - Independent Samples t-Test Results

| **Visual Property** | **Condition** | | ***t*** | ***df*** | ***p*** | **Cohen's d** |
| --- | --- | --- | --- | --- | --- | --- |
|  | **High consistency (Xiaomi)** | **Low consistency (Midea)** |  |  |  |  |
| Mean Luminance | 242.63 | 212.84 | 2.606 | 10.0 | 0.047 | 1.51 |
| Mean Entropy | 3.146 | 3.542 | -1.03 | 10.0 | 0.328 | -0.59 |
| *Note*: This table presents robustness check results based on independent samples t-tests (two-tailed). Cohen’s d is reported as a measure of effect size. | | | | | | |

**Supplementary Table S6**.Comparison of Primary and Robustness Check Results

| **Visual Property** | **Test** | **Statistic** | **Z/t** | **p** | **Effect Size** | **Interpretation** |
| --- | --- | --- | --- | --- | --- | --- |
| Luminance | Mann-Whitney U | U = 30.00 | 1.92 | 0.065 | r = 0.56 | Large effect, marginal significance |
|  | Independent t-test | t(10) = 2.61 | 2.61 | 0.047 | d = 1.51 | Large effect, significant |
| Visual Entropy | Mann-Whitney U | U = 12.00 | −0.96 | 0.394 | r = 0.28 | Small effect, non-significant |
|  | Independent t-test | t(10) = −1.03 | −1.03 | 0.328 | d = −0.59 | Medium effect, non-significant |
| *Note*. The primary analysis used Mann-Whitney U tests to maintain consistency with the nonparametric approach employed for experimental data. Robustness checks using independent samples t-tests yielded convergent conclusions for luminance (large effect in both tests) but divergent effect size estimates for visual entropy. | | | | | | |

**Supplementary Table S7.** Summary of HSV Color Distribution Characteristics of the Experimental Stimuli

| **Color Property** | **High Consistency (Xiaomi)** | **Low Consistency (Midea)** | **Interpretation** |
| --- | --- | --- | --- |
| Dominant hue composition | H_0_ (0°–5.625°): 89.25% of pixels | H_0_: 71.22%; secondary peaks at H₂ (red‑orange, 8.21%) and H₁₉ (blue, 4.30%) | Xiaomi exhibited a predominantly achromatic hue composition, whereas Midea showed a broader distribution of chromatic hues. |
| Hue distribution | Extremely concentrated in achromatic bin (89.25%); all other bins < 0.06 | 71.22% in achromatic bin; spread across red‑orange, yellow, blue, etc. | Hue values were more concentrated in Xiaomi and more broadly distributed across multiple hue categories in Midea. |
| Mean saturation | 3.55 | 15.87 | Xiaomi exhibited lower overall saturation than Midea. |
| Saturation distribution | 98.05% at S_0_ (lowest saturation); negligible elsewhere | 74.13% at S_0_; 20.9% across S₁–S₃₁ (medium to high saturation) | Saturation values were concentrated near the lower end in Xiaomi, whereas Midea showed a wider distribution across saturation levels. |
| Mean value (brightness) | 240.3 | 205.4 | Xiaomi exhibited a higher average brightness than Midea. |
| Value distribution | 64.77% at V_31_ (highest brightness); 28.7% in V₂₇–V₃₀ (high brightness) | 66.39% at V_31_; but 8–10% in low brightness (V₀–V₁₀) | Brightness values were more concentrated at the higher end in Xiaomi, whereas Midea displayed a broader distribution including more low-value pixels. |
| *Note*. Summary statistics are derived from normalized HSV histograms. H_0_–H_179_ denote hue histogram bins (0^o^–180°), whereas S_0_–S_255_ and V_0_–V_255_ denote saturation and value (brightness) histogram bins (0–255), respectively, following the OpenCV HSV representation. Full histogram distributions are presented in Supplementary Figure S1. | | | |
